# Linking Leaf Water Potential, Photosynthesis and Chlorophyll Loss With Mechanisms of Photo- and Antioxidant Protection in Juvenile Olive Trees Subjected to Severe Drought

**DOI:** 10.3389/fpls.2020.614144

**Published:** 2020-12-11

**Authors:** Sahar Baccari, Olfa Elloumi, Anissa Chaari-Rkhis, Erola Fenollosa, Melanie Morales, Noureddine Drira, Ferjani Ben Abdallah, Lotfi Fki, Sergi Munné-Bosch

**Affiliations:** ^1^Laboratoire LR16IO01, Institut de l’Olivier (IO), University of Sfax, Sfax, Tunisia; ^2^Laboratory of Plant Biotechnology (LR01ES21), Faculty of Sciences of Sfax, University of Sfax, Sfax, Tunisia; ^3^Department of Evolutionary Biology, Ecology and Environmental Sciences, Faculty of Biology, University of Barcelona, Barcelona, Spain; ^4^Laboratory of Plant Biodiversity and Dynamics of Ecosystems in Arid Area, Faculty of Sciences of Sfax, University of Sfax, Sfax, Tunisia

**Keywords:** drought tolerance, olive germplasm, photoprotection, α-tocopherol, xanthophyll cycle

## Abstract

The identification of drought-tolerant olive tree genotypes has become an urgent requirement to develop sustainable agriculture in dry lands. However, physiological markers linking drought tolerance with mechanistic effects operating at the cellular level are still lacking, in particular under severe stress, despite the urgent need to develop these tools in the current frame of global change. In this context, 1-year-old olive plants growing in the greenhouse and with a high intra-specific variability (using various genotypes obtained either from cuttings or seeds) were evaluated for drought tolerance under severe stress. Growth, plant water status, net photosynthesis rates, chlorophyll contents and the extent of photo- and antioxidant defenses (including the de-epoxidation state of the xanthophyll cycle, and the contents of carotenoids and vitamin E) were evaluated under well-watered conditions and severe stress (by withholding water for 60 days). Plants were able to continue photosynthesizing under severe stress, even at very low leaf water potential of −4 to −6 MPa. This ability was achieved, at least in part, by the activation of photo- and antioxidant mechanisms, including not only increased xanthophyll cycle de-epoxidation, but also enhanced α-tocopherol contents. “Zarrazi” (obtained from seeds) and “Chemlali” (obtained from cuttings) showed better performance under severe water stress compared to the other genotypes, which was associated to their ability to trigger a higher antioxidant protection. It is concluded that (i) drought tolerance among the various genotypes tested is associated with antioxidant protection in olive trees, (ii) the extent of xanthophyll cycle de-epoxidation is strongly inversely related to photosynthetic rates, and (iii) vitamin E accumulation is sharply induced upon severe chlorophyll degradation.

## Introduction

Olive tree (*Olea europaea* L.) is one of the most widespread crops in Mediterranean-type agroecosystems, with high economic and social importance. In semi-arid areas of the Mediterranean basin, the scarcity of precipitation combined with extreme climatic conditions such as high temperatures increase water stress in olive trees. It is particularly in this context that serious concerns have been raised about the impact of climate change on olive agricultural productivity. According to De Ollas et al. (2019), the Mediterranean region is expected to witness a decrease in the total amount of precipitation and an increase of the spread and intensity of drought. Among various countries from the Mediterranean basin, Tunisia is considered to be one of countries most exposed to climate change. During the drought episode of 1999–2002, the decrease of olive production reached 90%, which adversely affected farmers income (Gargouri et al., 2012). Considering this situation, much attention has recently been drawn to the valorization and conservation of plant genetic resources to achieve relatively high yields under severe drought. New approaches including the selection of drought-tolerant cultivars and rootstocks have been considered to reduce the effects of water stress. Several researchers have demonstrated that combinations of rootstocks and scions decrease the depressive effects of water stress and increase resistance to drought (Trifilo et al., 2007; Therios, 2009).

Understanding the mechanisms that olive trees have evolved to withstand drought stress is important for selecting drought-tolerant genotypes and improving olive yield and oil quality (Sofo et al., 2004). Under water stress conditions, olive trees regulate leaf water status by morphological, anatomical and physiological adaptations (Guerfel et al., 2009). Indeed, water deficit affects leaf water relations and gas exchange mechanisms of olive cultivars (Trabelsi et al., 2019). Some authors attributed the decrease of photosynthetic rate in response to water stress to stomatal conductance limitation (Perez-Martin et al., 2014; Ben Hamed et al., 2016). Stomatal control is an effective mechanism that decreases water loss through transpiration in drought conditions (Perez-Martin et al., 2014). When environmental stress factors become severe for plants, the mesophyll conductance and biochemical limitations further contribute to the decrease of photosynthetic activity (Flexas and Medrano, 2002).

The limitation of CO_2_ assimilation in water-stressed olive trees leads to an over-reduction of the photosynthetic electron chain (Sofo et al., 2004). Since chloroplasts cannot dissipate the excess light energy, there is a redirection of photon energy into processes that favor the production of reactive oxygen species (ROS) (Hasanuzzaman et al., 2017). High ROS concentration can seriously disrupt the metabolism of the plant causing enzyme inhibition, protein oxidation and membrane lipid peroxidation, and plants are endowed with antioxidant defense systems to protect the photosynthetic apparatus against oxidative damage (Sircelj et al., 2007). Several researchers have been interested in the enzymatic activity against oxidative stress in olive trees (Sofo et al., 2004; Ben Ahmed et al., 2009, Ben Abdallah et al., 2017), but little is known about the role of non-enzymatic antioxidants on the tolerance of olive trees under drought stress conditions. Among them, carotenoids and tocopherols constitute the main protection system against ROS, including their role in quenching the singlet oxygen (^1^O_2_) and in the case of tocopherols additionally preventing lipid peroxidation in thylakoid membranes (Muñoz and Munné-Bosch, 2019). The increase in carotenoids contents is one of the mechanisms evolved by the olive tree to protect the photosynthetic apparatus against photooxidation (Bacelar et al., 2007; Ben Abdallah et al., 2017). However, the identification of particular carotenoids involved in photo- and antioxidant protection under water deficit conditions in olive trees has been poorly investigated to date. The excess excitation energy is harmlessly dissipated in the antennae complexes of PSII as heat through a process which involves the xanthophyll cycle (Demmig-Adams and Adams III, 1996). Likewise, enhanced non-enzymatic antioxidant defense systems have been reported to improve plant abiotic stress tolerance in different studies (Munné-Bosch and Alegre, 2000; Hasanuzzaman et al., 2017). Some non-enzymatic antioxidant mechanisms, such as the increase in the content of phenolic compounds and glutathione, have been described to play a role in olive trees subjected to contrasting water availability regimes (Bacelar et al., 2006, 2007; Petridis et al., 2012), but to our knowledge no studies have been performed at the level of the xanthophyll cycle and α-tocopherol in olive trees, and even less these photo- and antioxidant mechanisms tested for their potential use in genotype selection.

Tunisian olive germplasm is characterized by a large number of varieties with about 200 cultivars. The main olive cultivars in Tunisia are Chemlali in the south and the center of the country and Chetoui in the north. These varieties account for 95% of the total olive trees and contribute more than 90% of the national production of olive oil. Chemlali is well known for its ability to cope with arid conditions (rainfall <200 mm per year). By taking advantage of the variability associated to the genotypic diversity of various young olive trees, we aimed here to unravel the link, if any, between leaf water potential, photosynthesis rates and chlorophyll loss with mechanisms of photo- and antioxidant protection in juvenile olive trees subjected to severe drought. Furthermore, it is discussed how the development of new photo- and antioxidant markers may be useful in order to select and develop new drought-tolerant olive tree cultivars for oil production in semi-arid regions. Due to the difficulty of performing studies in mature olive trees in field trials, we used here trees at an early stage of development. The juvenile phase of trees usually represents the most abiotic stress sensitive stage in woody plants (Shannon et al., 1994); therefore, young genotypes represented here may be considered a proper and valuable material for the detection of drought-tolerant genotypes. Furthermore, another important goal of our study was to establish new photo- and antioxidant markers that may be useful in order to select and develop new drought-tolerant olive tree cultivars in semi-arid regions. Selection methods including field trials testing several genotypes are generally limited, more specifically comparing rooted cutting with seedlings obtained from seeds (Laczko and Aghazarm, 2009). We aimed to fill this knowledge gap by testing the drought stress response of several olive trees at the juvenile stage under semi-controlled conditions to offer a rapid alternative (in comparison to field trials) in genotype selection for crop improvement.

## Materials and Methods

### Plant Material and Drought Stress Conditions

One-year-old olive trees (*Olea europaea* L.) were grown under natural daylight conditions with a mean maximum daily photosynthetically active radiation (PAR) of 1017 μmol m^–2^ s^–1^ in a plastic greenhouse at the Olive Tree Institute of Sfax. Plants were obtained from cuttings and/or from germination of seeds. Rooted cuttings were obtained from the two main Tunisian olive cultivars “Chemlali” and “Chetoui,” while seedlings were regenerated from the seeds of wild olive tree “Oleaster” and from three olive cultivars “Chetoui,” “Chemlali” and “Zarrazi” which are cultivated, respectively, in the north, the center and the south of Tunisia. All seeds were collected from the experimental station “Taous” of the Olive Tree Institute (central Tunisia, 34°56′38″N, 10°36′50″E), except for the wild olive tree “Oleaster,” whose seeds were collected from the National Park of Ichkeul (north Tunisia, 37°10′00″N, 9°40′00″E).

After germination, seedlings were selected for homogeneity and grown for 3 months on an MS medium (Murashigue and Skoog, 1962) supplemented with 8 g⋅L^–1^ agar and 30 g⋅L^–1^ sucrose. Both seedlings and rooted cuttings were transplanted in 1-L plastic pots filled with sand and peat (2:1, v/v) and then irrigated twice a week with half-strength Hoagland”s solution (Hoagland and Arnon, 1950) for 8 months. During this period, mean daily maximum and minimum temperatures and relative humidity in the greenhouse ranged from 20 to 30°C, and between 40 and 50%, respectively. Before stress application, soil field capacity was calculated. Indeed, all the plants were saturated with water and left to drain freely until drainage became negligible. After 24 h, the weight recorded for each individual pot (W1) was considered as the soil field capacity. Soil water content was monitored by weighing all the pots every 2 days (W2) and the amount of water transpired was determined and restored by irrigation. The weight difference (W1–W2) allowed a calculation of the amount of water lost by transpiration and used for irrigation. Drought stress was imposed by a gradual reduction of 20% of the total transpired water every 2 days until full irrigation withdrawal. Well-watered (control) plants were irrigated with 100% of soil field capacity throughout the experiment.

Plants were arranged in two completely randomized blocks design. Each block represents a water regime: irrigated and stressed. A total number of 24 seedlings and 3 plants per cultivar were selected for the irrigated water regime. For the contrasting water regime, a total number of 50 seedlings and 8 plants per cultivar were used. Measurements were performed on plants exposed to severe stress after 60 days of starting treatments, including leaf water potential, leaf gas exchange and mechanisms of photo- and antioxidant protection. Growth parameters were estimated both after 30 and 60 days of starting treatments, For biochemical analyses, fully-expanded young leaves exposed to sunlight were sampled between 10 a.m. and 11 a.m. (local time), immediately frozen in liquid nitrogen and stored at −20°C until analyses.

### Growth Parameters

Stem elongation (cm) and the number of leaves per plant were measured for each plant at the beginning of stress (*H*_*i*_, *L*_*i*_), at 30 days (*H*_30_, *L*_30_) and at 60 days (*H*_60_, *L*_60_) of drought. Stem growth rate (SGR) and leaf production rate (LPR) were estimated from these parameters according to the equations described by Sun et al. (2019) and Rodrigues de Lima et al. (2019).

### Leaf Water Potential and Leaf Gas Exchange

Leaf water potential (Ψ_*L*_) was measured using a Scholander pressure chamber model SKPM 1400 (Skye Instruments, Powys, United Kingdom) between 9:00 and 11:00 local time. Net photosynthesis rate (A_*n*_), stomatal conductance (gs), transpiration (E), water use efficiency (A/E) and intercellular to ambient carbon dioxide concentration ratio (Ci/Ca) were determined between 9:00 and 11:00 using a portable gas exchange system (Cl-340 Handheld Photosynthesis System). During leaf gas exchange measurements, photosynthetically-active radiation was set at 1250 μmol⋅m^–2^⋅s^–1^, leaf temperature at 35°C, CO_2_ concentration at 430 ppm and vapor pressure deficit at 5 KPa. All measurements were carried out on fully-expanded young leaves taken from the median part of the shoot. Leaf water potential determination was performed using the whole leaf with the petiole, taking care the latter remained intact during measurements.

### Chlorophyll Contents and Extent of Photo- and Antioxidant Protection

The extraction and HPLC analysis of chlorophylls and carotenoids were performed as described by Munné-Bosch and Alegre (2000). In short, leaves were ground in liquid nitrogen and pigments extracted with cold methanol using ultrasonication. After centrifuging at 1000 × *g* for 10 min at 4°C, the supernatant was collected, and the pellet re-extracted with the same solvent until it turned colorless. Then, supernatants were pooled and filtered with a 0.22-μm pore size. Pigments were separated by high-performance liquid chromatography (HPLC) on a Dupont non-end capped Zorbax ODS-5-μm column (250 mm long, 4.6 mm i.d.; 20% carbon, Teknokroma, Sant Cugat, Spain) at 30°C at a flow rate of 1 ml⋅min^–1^ for 38 min. The solvent mixture for the gradient, detection at 445 nm (diode array detector, HP1100 Series HPLC System, Agilent Technologies, Santa Clara, CA, United States) and calculation of pmol/are ratios for quantification of chlorophyll a, chlorophyll b, neoxanthin, violaxanthin, antheraxanthin, zeaxanthin, lutein, and β-carotene were performed as described by Munné-Bosch and Alegre (2000). The de-epoxidation state of the xanthophyll cycle (DPS) was calculated as DPS = (Z + 0.5A)/(V + Z + A), where V, A, and Z are violaxanthin, antheraxanthin and zeaxanthin, respectively.

The same methanolic extract was used for the determination of tocopherols, which were separated isocratically on a normal-phase HPLC system (JASCO, Tokyo, Japan) and quantified using a fluorescent detector as described by Amaral et al. (2005). Detection was carried out at an excitation of 295 nm and emission at 330 nm. α-Tocopherol was quantified by co-elution with an authentic standard (Sigma–Aldrich, Steinheim, Germany) and quantified using a calibration curve. From all vitamin E homologs measured (α-, β-, γ-, and δ-tocopherol), only the α-tocopherol form accumulated in olive tree leaves.

### Statistical Analysis

Statistical analyses were carried out with the SPSS Base 20.0 software (Chicago, IL, United States). A two-way analysis of variance (ANOVA) was used to examine cultivar and treatment effects. Duncan”s multiple range tests were used for mean comparison at *p* ≤ 0.05. Correlation between variables was determined according to Pearson tests.

## Results

### Impact of Drought on Growth, Leaf Water Potential and Photosynthesis

**FIGURE 1:**
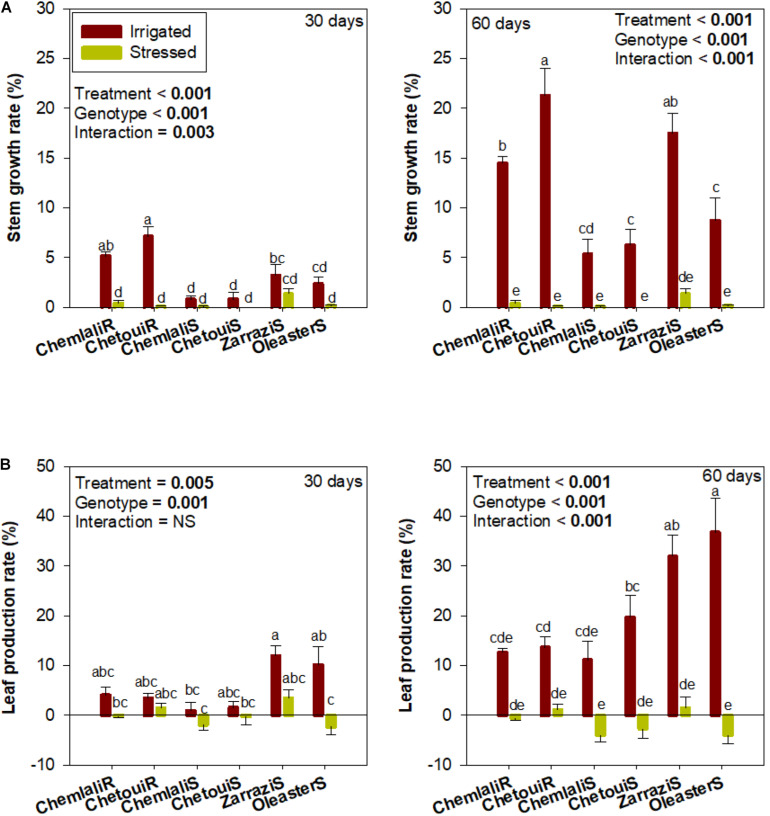
Stem and leaf growth in olive trees subjected to mild and severe drought stress. Effects of drought stress periods (30 and 60 days after start of treatments) on **(A)** stem growth and **(B)** leaf production rate in olive trees obtained from cuttings (cv. “Chemlali” and “Chetoui”) and obtained from seeds (“Chemlali S,” “Chetoui S,” “Zarrazi S” and “Oleaster S”). Values represent means ± S.E. of *n* ≥ 3 for irrigated plants and *n* ≥ 8 for water-stressed plants. A two-way analysis of variance (ANOVA) was used to examine if the effects of treatment (water regime) and genotype (cultivar) were statistically significant and to look at the significance of interaction effects. Different letters indicate significant differences between genotypes in each condition using Duncan *post hoc* tests (*p* ≤ 0.05).

Drought and cultivar effects on growth were observed (Figure 1A). Leaf production rate (LPR) dropped severely in all olive trees exposed to drought stress, with the seedlings “Chemlali” (“Chemlali S”) and “Oleaster” (“Oleaster S”) being the most affected already at 30 days of drought (Figure 1B). Increasing drought stress up to 60 days resulted in a further leaf production decrease with a response that depended on the cultivar, as evidenced by a strong cultivar × water regime interaction (*p* < 0.001) (Figure 1B).

**FIGURE 2:**
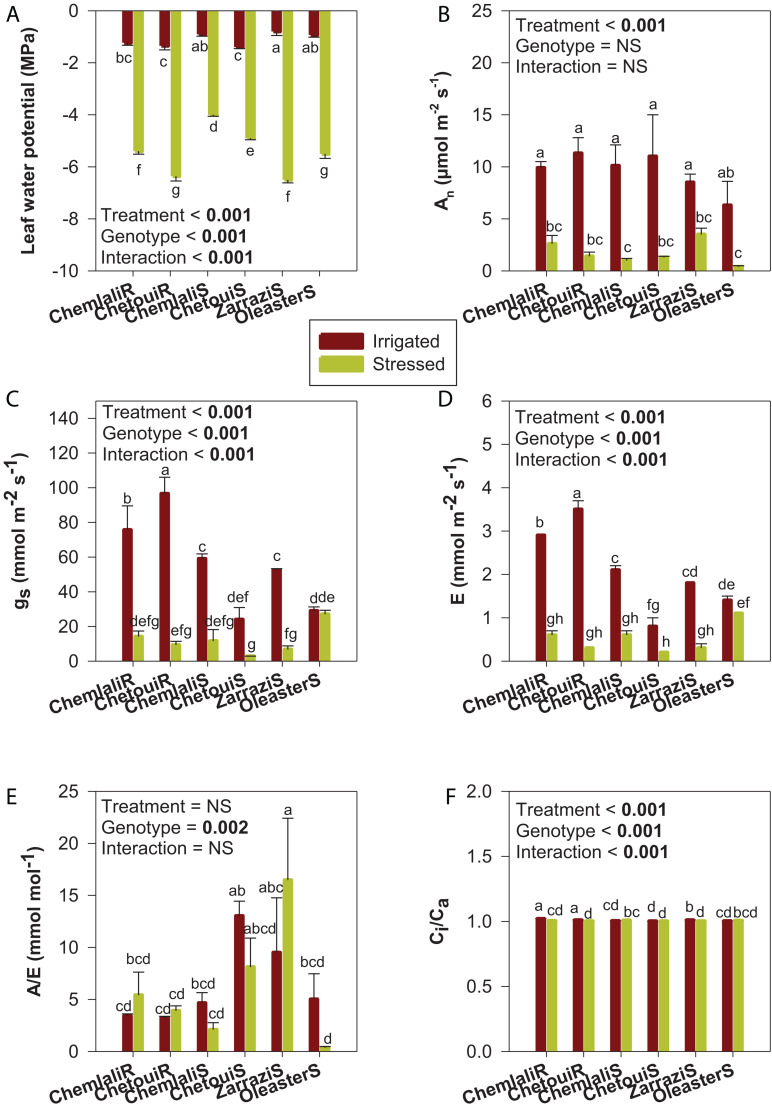
Water status and leaf gas exchange in olive trees subjected to severe drought stress. **(A)** Leaf water potential (Ψ_*L*_), **(B)** net photosynthesis rate (An), **(C)** stomatal conductance (gs), **(D)** transpiration (E), **(E)** water use efficiency (A/E) and **(F)** intercellular to ambient carbon dioxide concentration ratio (Ci/Ca) in olive trees obtained from cuttings (cv. “Chemlali” and “Chetoui”) and obtained from seeds (“Chemlali S,” “Chetoui S,” “Zarrazi S” and “Oleaster S”) after 60 days of drought stress. Values represent means ± S.E. of *n* = 3. A two-way analysis of variance (ANOVA) was used to examine if the effects of treatment (water regime) and genotype (cultivar) were statistically significant and to look at the significance of water regime × cultivar interaction effects. Means having the same letter are not significantly different according to Duncan *post hoc* test at *p* ≤ 0.05.

In irrigated conditions, Ψ_*L*_ showed the lowest values (−1.4 MPa) in both “Chetoui” rooted cuttings and seedlings, which significantly differ to other genotypes except “Chemlali” (Figure 2A). The highest values (−0.8 MPa) were observed for “Zarrazi” seedlings. For the stressed treatment, Ψ_*L*_ decreased significantly (*p* < 0.001) in all olive trees compared to control plants (Figure 2A). The response to water stress depended on the cultivar as evidenced by cultivar × water regime interaction (*p* < 0.001). The reduction of Ψ_*L*_ ranged from 71 to 88%.

A_*n*_ decreased significantly after 60 days of drought stress in all genotypes, irrespective of whether the plants were obtained from cuttings or seeds (Figure 2B). As compared to the control plants, “Zarrazi” seedlings (“Zarrazi S”), followed by “Chemlali” rooted cuttings, were the less affected by water deficit. For the rest of olive trees, more severe reductions due to water deficit were observed, ranging from 87 to 93% relative to their respective controls.

Except for “Oleaster” seedlings, the high decrease of A_*n*_ coincided with the decrease in gs and E. Stressed olive plants exhibited severe decreases in gs and E, as compared to well-watered plants. However, for the “Oleaster S,” despite the decrease in photosynthetic activity (more than 92%), no significant difference was observed in gs and E between irrigated and stressed plants (Figures 2C,D). Water use efficiency (calculated as A/E) did not show significant differences between irrigated and stressed olive trees, with the exception of “Oleaster S,” which showed a severe reduction in this stress marker. By contrast, “Zarrazi S” was the most efficient in water use (Figure 2E). The intercellular to ambient carbon dioxide concentration ratio (Ci/Ca) kept nearly constant in response to drought with values close to 1 for all genotypes (Figure 2F).

### Impact of Drought Stress on Photo- and Antioxidant Protection

A significant interaction between cultivar and water regime (*p* = 0.001) was recorded on leaf chlorophyll (Chl a + b) content after 60 days of drought stress (Table 1). Compared to control plants, withholding irrigation induced a significant decrease in Chl a + b, with the exception of “Zarrazi S” and both rooted cuttings “Chemlali” and “Chetoui” in which no significant changes were observed. The highest decrease of Chl a + b was recorded in “Oleaster S” which was about 36.4%. Also, no significant change in terms of chlorophyll a/b ratio (Chl a/b) was observed between irrigated and stressed olive trees, with the exception of “Chemlali S” and “Chetoui” rooted cuttings for which significant decreases of 12.2 and 10.2%, respectively, were obtained (Table 1).

**TABLE 1:** Contents of chlorophyll (Chl a + b mg⋅g^–1^ DW), chlorophyll a/b ratio (Chl a/b), xanthophyll cycle pool per unit of Chl (VZA/Chl mmol⋅mol^–1^), de-epoxidation state of the xanthophyll cycle (DPS), lutein per Chl ratio (Lut/Chl mmol⋅mol^–1^), β-carotene per Chl ratio (β-car/Chl mmol⋅mol^–1^), and α-tocopherol per unit of Chl (α-Toc/Chl mmol⋅mol^–1^) in olive trees under irrigated and water stress conditions for 60 days.

			Chla + b	Chl a/b	VZA/Chl	DPS	Lut/Chl	β-car/Chl	α-Toc/Chl
Cuttings	“Chemlali”	Irrigated	5.5 ± 0.5^de^	1.9 ± 0.02^ab^	269.7 ± 21.3^cde^	0.48 ± 0.01^b^	573.3 ± 28.2^bc^	193.8 ± 9.5^ab^	40.0 ± 1.4^bc^
		Stressed	4.6 ± 0.0^ef^	1.8 ± 0.05^bcd^	383.0 ± 26.6^a^	0.65 ± 0.00^a^	565.9 ± 18.8^bc^	109.7 ± 28.8^c^	82.5 ± 6.6^a^
	“Chetoui”	Irrigated	4.8 ± 0.1^ef^	2.0 ± 0.02^a^	204.2 ± 7.2^e^	0.50 ± 0.02^b^	460.3 ± 5.0^d^	182.1 ± 11.9^b^	60.6 ± 8.5^ab^
		Stressed	4.0 ± 0.1^f^	1.8 ± 0.03^bcd^	324.0 ± 39.2^abc^	0.66 ± 0.02^a^	531.6 ± 14.4^bcd^	52.5 ± 10.9^c^	85.9 ± 26.4^a^
Seedlings	“Chemlali S”	Irrigated	8.9 ± 0.7^b^	1.8 ± 0.01^bcd^	200.2 ± 19.8^e^	0.45 ± 0.04^b^	562.0 ± 41.0^bc^	217 ± 25.5^ab^	14.9 ± 0.6^def^
		Stressed	5.8 ± 0.1^de^	1.6 ± 0.04^e^	366.9 ± 37.3^ab^	0.65 ± 0.00^a^	678.6 ± 38.0^a^	105.7 ± 10.8^c^	25.4 ± 10.9^cde^
	“Chetoui S”	Irrigated	8.7 ± 0.5^b^	1.9 ± 0.01^ab^	217.2 ± 18.4^e^	0.49 ± 0.02^b^	512.5 ± 24.4^cd^	204.2 ± 19.3^ab^	9.41 ± 1.7^ef^
		Stressed	6.1 ± 0.4^de^	1.9 ± 0.10^abc^	332.9 ± 22.1^abc^	0.61 ± 0.01^a^	604.0 ± 16.8^abc^	110.7 ± 39.9^c^	57.1 ± 17.8^ab^
	“Zarrazi S”	Irrigated	7.7 ± 0.6^bc^	1.9 ± 0.04^abc^	256.8 ± 21.3^cde^	0.45 ± 0.03^b^	621.3 ± 50.6^ab^	247.9 ± 14.2^a^	11.4 ± 2.8^ef^
		Stressed	6.5 ± 0.5^cd^	1.8 ± 0.06^bcd^	399.0 ± 19.9^a^	0.66 ± 0.00^a^	616.5 ± 26.3^ab^	180.2 ± 9.7^b^	35.0 ± 5.1^bcd^
	“Oleaster S”	Irrigated	12.9 ± 0.5^a^	1.7 ± 0.05^cde^	227.7 ± 7.23^de^	0.48 ± 0.01^b^	532.3 ± 24.7^bcd^	172.3 ± 8.1^b^	5.5 ± 0.6^f^
		Stressed	8.2 ± 0.4^b^	1.7 ± 0.06^de^	302.3 ± 34.6^bcd^	0.62 ± 0.01^a^	576.0 ± 12.6^bc^	71.2 ± 16.5^c^	15.0 ± 2.8^def^
	Treatment		***p* < 0.001**	***p* < 0.001**	***p* < 0.001**	***p* < 0.001**	**0.003**	***p* < 0.001**	0.907
	Genotype		***p* < 0.001**	**0.001**	**0.032**	0.668	**0.001**	***p* < 0.001**	***P* < 0.001**
	Interaction		**0.001**	0.242	0.58	0.353	0.173	0.697	0.931

*Values represent means ± S.E. of *n* = 4. A two-way analysis of variance (ANOVA) was used to examine if the effects of treatment (water regime) and genotype (cultivar) were statistically significant and to look at the significance of interaction effects. Means on the same column having a same letter are not significantly different according to Duncan *post hoc* test at *p* ≤ 0.05. Bold values show significant differences.*

Drought stress increased significantly the xanthophyll cycle pool expressed per unit of chlorophyll (VZA/Chl) and the de-epoxidation state of the xanthophyll cycle (DPS) in olive trees compared to control plants (*p* < 0.001). The increase in VZA/Chl ranged in all genotypes between 1.3 and 1.9-fold in stressed plants compared to their respective controls. The highest VZA/Chl ratio was recorded in “Zarrazi S,” followed by “Chemlali” rooted cuttings, whereas the lowest one was obtained for “Oleaster S” (Table 1). An effect of water regime, but not of cultivar, was also observed in DPS (*p* < 0.001). The stress-related increase in DPS ranged between 1.2 and 1.5-fold relative to controls, respectively, in “Chetoui” seedlings (“Chetoui S”) and “Zarrazi S.” In contrast, the β-carotene per unit of chlorophyll (β-car/Chl) decreased significantly under stress conditions in all olive trees with “Zarrazi S” showing the lowest decrease (27.2%). However, the lutein to Chl ratio (Lut/Chl) increased significantly with stress in “Chemlali S “and remained unchanged in the rest of the olive trees compared to control plants (Table 1).

Under water stress conditions, α-tocopherol per unit of chlorophyll (α-Toc/Chl) increased significantly in stressed olive trees compared to control plants, with the exception of “Chemlali S,” “Oleaster S” and “Chetoui” rooted cuttings, in which no significant changes were observed (Table 1). This increase ranged between 2 and 6-fold relative to controls in “Chemlali” rooted cuttings and “Chetoui S,” respectively.

**FIGURE 3:**
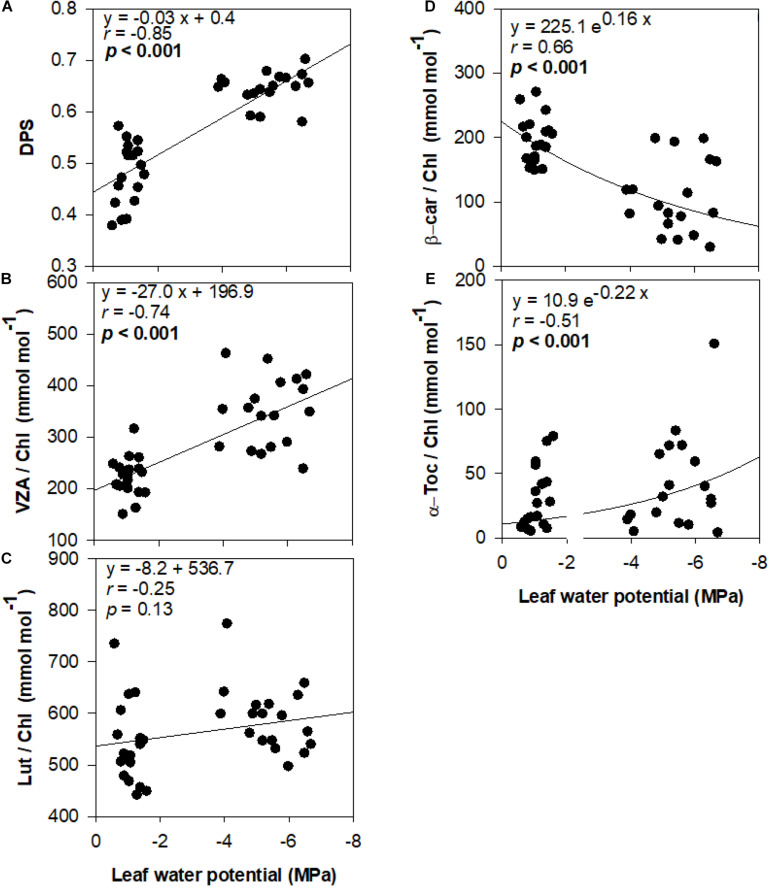
Relationship between leaf water status and photo- and antioxidant protection in olive trees. Relationship between leaf water potential and the de-epoxidation state of the xanthophyll cycle **(A)**, the contents of the xanthophyll cycle pool **(B)**, lutein **(C)**, β-carotene **(D)**, and α-tocopherol **(E)** per unit of chlorophyll of olive trees after 60 days of treatments. Correlation was determined according to Pearson test. *p* values and corresponding correlation coefficient (*r*) are shown.

**FIGURE 4:**
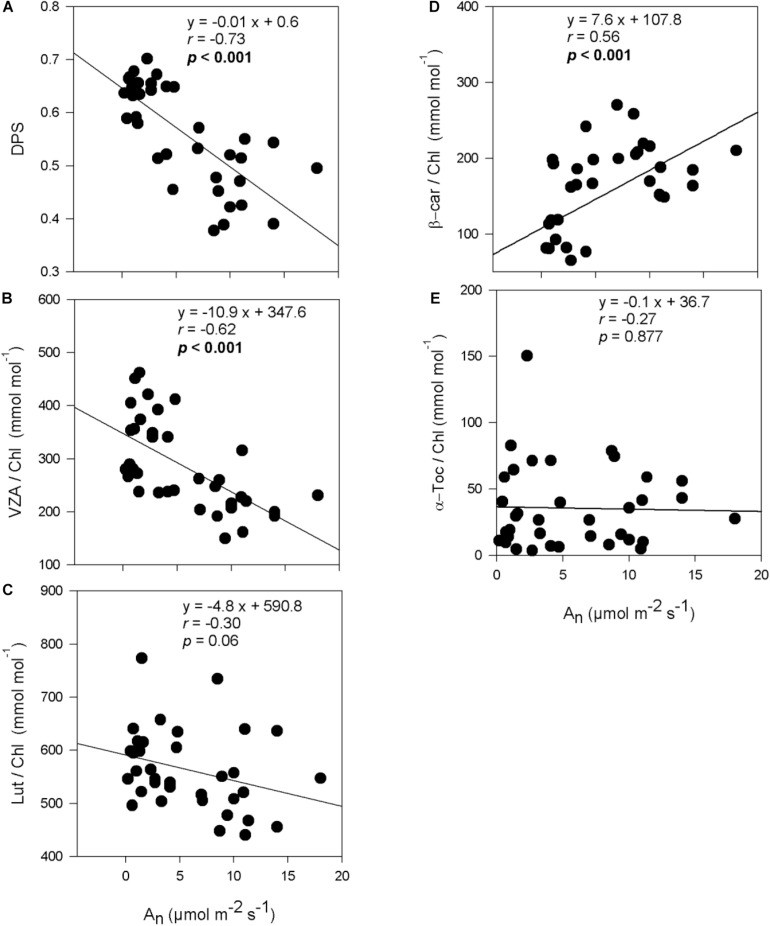
Relationship between photosynthesis and photo- and antioxidant protection in olive trees. Relationship between net photosynthesis rates and the de-epoxidation state of the xanthophyll cycle **(A)**, the contents of the xanthophyll cycle pool **(B)**, lutein **(C)**, β-carotene **(D)**, and α-tocopherol **(E)** per unit of chlorophyll of olive trees after 60 days of treatments. Correlation was determined according to Pearson test. *p* values and corresponding correlation coefficient (r) are shown.

**FIGURE 5:**
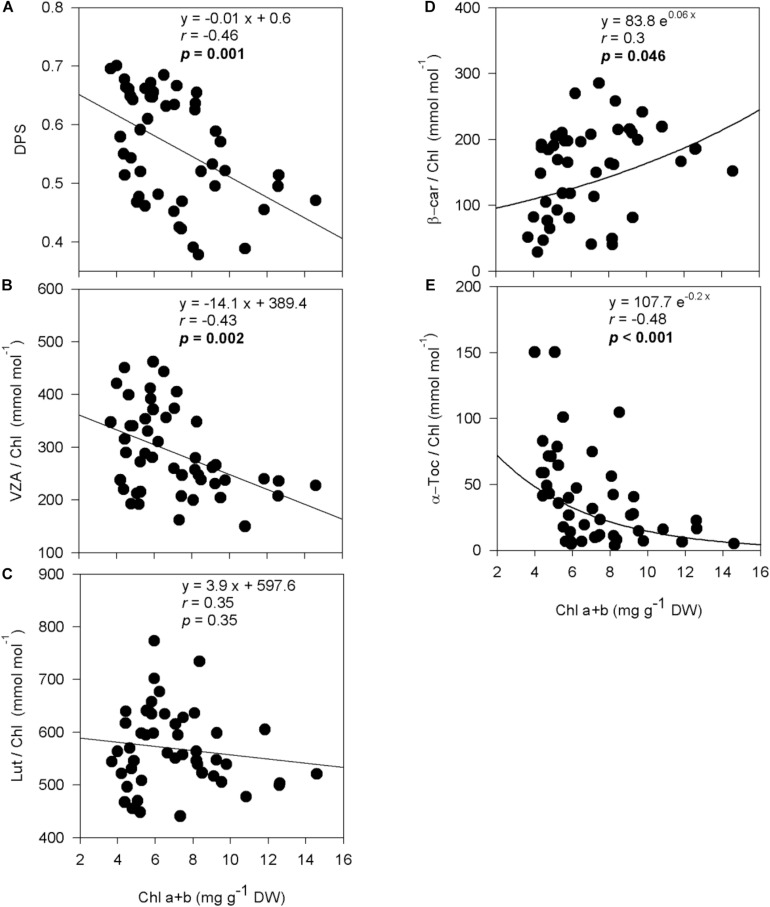
Relationship between chlorophyll loss and photo- and antioxidant protection in olive trees. Relationship between chlorophyll contents and the de-epoxidation state of the xanthophyll cycle **(A)**, the contents of the xanthophyll cycle pool **(B)**, lutein **(C)**, β-carotene **(D)**, and α-tocopherol **(E)** per unit of chlorophyll of olive trees after 60 days of treatments. Correlation was determined according to Pearson test. *p* values and corresponding correlation coefficient (r) are shown.

De-epoxidation state of the xanthophyll cycle and VZA levels were closely related to leaf water potential (Figure 3), photosynthetic activity (Figure 4) and leaf chlorophyll content (Figure 5). In fact, results showed negative correlations between either DPS or VZA with Ψ_*L*_ (*r* = −0.85 and *r* = −0.74, respectively), A_*n*_ (*r* = −0.73 and *r* = −0.62, respectively), Chl a + b (*r* = −0.46 and *r* = −0.43, respectively). It appears that a linear and significant increase of DPS and VZA levels occurred when the Ψ_*L*_ decreased below −4 MPa and A_*n*_ was reduced below 5 μmol CO_2_⋅m^–2^⋅ s^–1^. Moreover, a significant correlation was observed between Ψ_*L*_ and α-Toc/Chl, and between Chl a + b and α-Toc/Chl (*r* = −0.51 and *r* = −0.48, respectively, Figures 3–5).

## Discussion

The effects of water stress on growth may be considered the first line of defense, as it has been previously reported on olive trees (Trabelsi et al., 2019). This is essentially due to the inhibition of cell elongation by the interruption of water flow from the xylem to the surrounding cells (Nonami, 1998) and serves to reduce the amount to total water transpired by the plant under drought conditions. In the present study, growth measurements revealed genotypic variability in response to drought stress. Using stem and leaf growth parameters as indicators of tolerance, “Chemlali” rooted cuttings and “Zarrazi S” can be considered the genotypes more tolerant to drought. In contrast, “Chemlali S,” “Oleaster S” and “Chetoui S” can be considered the most susceptible to drought stress because these seedlings showed the highest reduction in LPR. The superior performance of “Zarrazi S” to drought stress could be explained by the genotypic adaptation to the local environment of this variety, which is natural from Medenine - southern Tunisia, in which the average annual rainfall does not exceed 180 mm. In our study, two irrigation treatments and 60 days of drought stress were selected for measurements of leaf water potential, net photosynthesis and photo- and antioxidant protection, which due to the high tolerance of some of the genotypes tested led to an adequate comparison between genotypes under well-watered and severe water deficit conditions. No information is available in the literature on selection methods including photoprotection and chloroplastic antioxidant markers such as carotenoids and tocopherols, and even less comparing olive tree rooted cuttings with seedlings (obtained from seeds). We filled here this knowledge gap by testing the drought stress response of several olive trees at the juvenile stage under semi-controlled conditions to offer a rapid alternative (in comparison to field trials) in genotype selection for crop improvement. Furthermore, found a link between both water potential and net photosynthesis rates with various photo- and antioxidant markers in olive trees subjected to severe water deficit, which helps us better understand the response of a very tolerant xerophyte plant, such as olive trees, to severe drought.

Plant growth decrease is mainly due to the loss of turgor pressure (Nonami, 1998). Indeed, exposure of plants to severe drought stress (60 days without irrigation) decreased significantly the olive trees water status attaining leaf water potential values between −6 and −4 MPa, thus indicating severe water deficit. For olive trees (cv. Koroneiki), 30 days without watering decreased significantly the Ψ_*L*_ to achieve −6.5 MPa (Boussadia et al., 2008). Similarly, for pistachio, Behboudian et al. (1986) revealed very low leaf water potential (−6 MPa), which is not completely unusual for woody plants growing under severe drought conditions. Furthermore, our results indicated that Ψ_*L*_ were differently affected by drought stress depending on the cultivar. Contrary to “Zarrazi S,” “Chetoui S” showed the less reduction rate (71.4%) of Ψ_*L*_. This difference is probably associated with the different mechanisms of osmotic adjustment adopted by the olive trees under water stress conditions, as previously reported by Dichio et al. (2005). In olive trees, damage caused by drought stress is mainly associated with a decrease of photosynthesis activity. The net photosynthesis rate showed a significant decrease (between 58 and 93%) after a long period of drought stress (60 days). These differences in gas exchange responses between olive trees could be used as a selection factor for olive trees grown under drought conditions (Ben Ahmed et al., 2009; Guerfel et al., 2009). In fact, the lowest decrease of A_*n*_ was recorded in “Zarrazi S” and, in a second degree, in “Chemlali” rooted cuttings, which allows them to withstand water stress more effectively than the other tested olive trees. These results are in accordance with those found by Guerfel et al. (2009), who have indicated a small decrease in A_*n*_ in “Chemlali” classified as a drought-tolerant cultivar. However, “Oleaster S” can be considered the most sensitive to water stress as they showed the highest depressive effect of water stress on A_*n*_ (92.8% reduction compared to control). In susceptibility to water stress, “Oleaster S” was followed by “Chemlali S,” “Chetoui S” and “Chetoui” rooted cuttings with a mean of 88.4% reduction on A_*n*_ compared to its respective control. Moreover, the increase in water use efficiency (calculated here as the A/E ratio) could be considered as a drought tolerance index (Jimenez et al., 2013;,  Santana-Vieira et al. 2016). As compared to the rest of olive trees, “Zarrazi S” were able to maintain the highest A_*n*_ value even with a minimum Ψ_*L*_ of about −6.5 MPa and was more efficient in water use under severe drought conditions. Plants with a high A/E ratio may be suitable for cultivation in semi-arid regions (Santana-Vieira et al., 2016). It should be noted that cultivated varieties “Chemlali” and “Chetoui” are at the origin of selected genotypes propagated vegetatively. The “Chemlali” variety is known for its tolerance to drought, hence the predominance of its cultivation in central and southern Tunisia in arid and semi-arid conditions, whereas the “Chetoui” variety with less pronounced tolerance to drought predominates in the north with climatic conditions where rainfalls are more abundant.

It is interesting to note that despite the very low values of −6 MPa achieved, some photosynthetic activity was maintained in some cultivars of olive trees. At leaf water potentials below −4 MPa, net photosynthesis rate ranged between 0.45 and 3.57 μmol CO_2_⋅m^–2^⋅s^–1^. The ability to maintain some photosynthetic activity at very low leaf water potential was reported in other fruity tree species such as pistachio (Behboudian et al., 1986). Indeed, these authors showed that although photosynthesis declined with decreasing leaf water potential, plants continued to photosynthesize until a leaf water potential of as low as −5 MPa was reached which is a typical response of xerophytic plants. Likewise, in the case of olive tree (cv. Koroneiki), Boussadia et al. (2008) recorded a net CO_2_ assimilation rate of 1 μmol CO_2_⋅m^–2^⋅s^–1^ under severe drought stress in which the leaf water potential dropped to very low values of −6.5 MPa. It appears from the present study that stressed young olive plants maintained some photosynthetic activity at low leaf water potential, which might allow them help surviving the severe water stress conditions. It is noteworthy that conditions imposed to plants during leaf gas exchange measurements were very severe, including high temperatures (35°C) and a high vapor pressure deficit (5 KPa), which explains the low stomatal conductance and photosynthesis rates together with the high Ci/Ca values observed in well-watered plants. Similar stomatal conductance, net photosynthesis rates and Ci/Ca ratios were observed for anti-sense Rubisco transgenic tobacco plants (Von Caemmerer et al., 2004). Leaf gas exchange results obtained in our study suggest that there was a stomatal and non-stomatal limitation of photosynthesis under well-watered conditions, and that both of them increased under severe water deficit, given that not only stomatal conductance and net photosynthesis were reduced but also the Ci/Ca ratio kept constant in water-stressed plants. It is very likely that the severe stomatal closure in severely water-stressed plants did not result in an increased Ci/Ca ratio because Rubisco and non-stomatal limitations of photosynthesis impaired the correct functioning of the Calvin cycle, which might indeed led to a oversaturation of the photosynthetic electron transport and excess excitation energy in chloroplasts. To avoid the potential deleterious effects of oxidative damage induced by water stress, plants have evolved both enzymatic and non-enzymatic antioxidant defense systems. The role of photosynthetic pigments (total carotenoids) and enzymatic antioxidant systems as mechanisms of water stress tolerance in olive trees have been already investigated previously (Sofo et al., 2005, 2008; Bacelar et al., 2006, 2007; Brito et al., 2009; Ben Abdallah et al., 2017; reviewed by Brito et al., 2019). Hence, we focused here on the study of specific photosynthetic pigments with a function in photoprotection (such as individual xanthophylls and carotenes) and other non-enzymatic chloroplastic antioxidants (such as tocopherols) Among those molecules, carotenoids and tocopherols are well known to play an important role in preventing photo-oxidative stress in several species, including other xerophytes such as rosemary plants (Munné-Bosch and Alegre, 2000). Our results showed an increase in the xanthophyll cycle pool (VZA) and the de-epoxidation state of the xanthophyll cycle (DPS) levels in stressed olive trees. The contribution of VZA to olive drought tolerance mechanisms is clearly demonstrated in “Zarrazi S” and “Chemlali” rooted cuttings that accumulated the largest amount of VZA/Chl (399 and 383 mmol⋅mol^–1^, respectively). The lowest VZA/Chl level (302 mmol⋅mol^–1^) was recorded in “Oleaster S.” Indeed, water stress is known to induce the activation of the xanthophyll cycle, in which violaxanthin is de-epoxidized to antheraxanthin and then zeaxanthin, enabling excess light energy to be dissipated as heat (Demmig-Adams and Adams III, 1996). The increase observed in DPS and VZA/Chl levels was associated with the decrease in Ψ_*L*_ and A_*n*_ (Figures 3, 4). It appears therefore that the antioxidant defense mechanisms can be efficiently developed even when the Ψ_*L*_ decreased below −4 MPa and A_*n*_ turned less than 5 μmol CO_2_⋅m^–2^⋅s^–1^. Indeed, loss of leaf turgor and photosynthetic limitation might be involved in triggering the activation of photoprotective mechanisms in young olive trees under severe water stress. β-carotene/Chl ratio was significantly decreased under drought conditions. A similar response was reported by Sircelj et al. (1999), who described an increase in the zeaxanthin level at the expense of β-carotene in field-grown apple trees subjected to water stress. Reduced β-carotene associated with increased zeaxanthin contents, as occurred in our study, may be indicative of an enhanced conversion of β-carotene to xanthophyll cycle components for excess energy dissipation, as well as an increased oxidation of β-carotene by singlet oxygen in light harvesting complexes and thylakoid membranes (Burton and Ingold, 1984; D’Alessandro and Havaux, 2019). Furthermore, changes in α-tocopherol content under water stress conditions were also observed. In fact, Ψ_*L*_ decreases paralleled an accumulation of α-Toc/Chl in leaves in all studied cultivars, although significantly only for “Chemlali” rooted cuttings, “Chetoui S” and “Zarrazi S.” Differences observed in α-Toc/Chl level could be related to genotypic differences in antioxidant capacity among olive trees. α-Tocopherol is involved in the elimination of ^1^O_2_ (Fahrenholtz et al., 1974; Muñoz and Munné-Bosch, 2019) and among other functions, α-tocopherol is known to inhibit the propagation of lipid peroxidation in thylakoid membranes by reducing the accumulation of lipid peroxyl radicals in chloroplasts (Munné-Bosch and Alegre, 2002; Muñoz and Munné-Bosch, 2019). α-Toc/Chl ratio was also associated with the decrease in Chl a + b, which may be related to the fact that this antioxidant can be synthesized using the phytol recycling pathway from chlorophyll degradation (in the so-called phytol recycling pathway, see Muñoz and Munné-Bosch, 2019). The accumulation of photoprotective molecules together with a reduction in chlorophyll content may be an effective strategy to survive severe drought conditions, as it has been observed in other highly drought-tolerant plants (Kyparissis et al., 2000; Munné-Bosch and Alegre, 2000).

**FIGURE 6:**
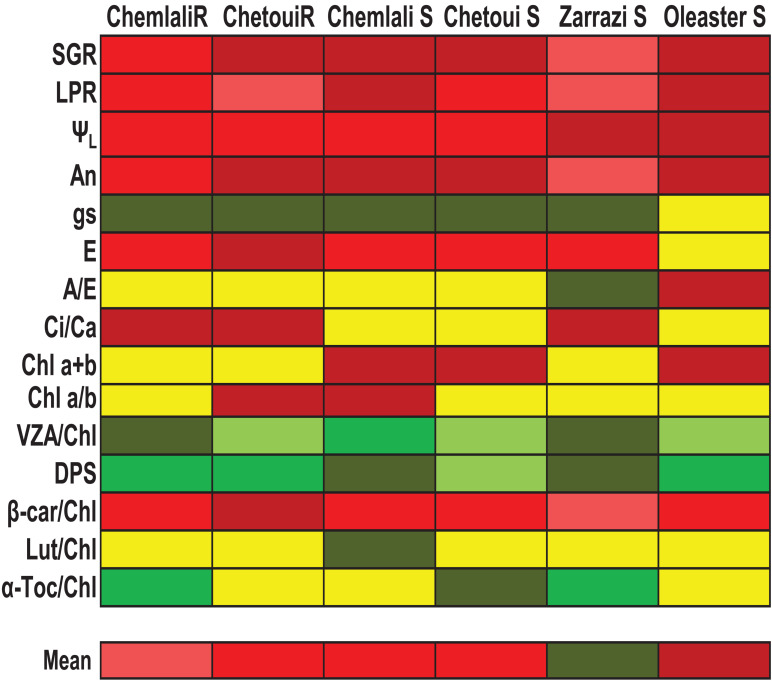
Tolerance to severe water stress of olive genotypes. The color gradation ranges from dark red to dark green to indicate progressively increased stress tolerance. Three levels of susceptibility and three levels of tolerance were given for each of the parameters in red and green, respectively, and the mean of tolerance was then calculated considering all parameters measured after 60 days of treatments. SGR, Stem growth rate; LPR, Leaf production rate; Ψ_*L*,_ Leaf water potential; A_*n*_, Net photosynthesis rate; gs, stomatal conductance; E, transpiration; A/E, water use efficiency; Ci/Ca, intercellular to air carbon dioxide concentration ratio; Chl a + b, Total chlorophyll; Chl a/b, chlorophyll a/b ratio; VZA/Chl, xanthophyll cycle pool per unit of Chl; DPS, de-epoxidation state of the xanthophyll cycle; Lut/Chl, lutein per Chl ratio; β-car/Chl, β-carotene per Chl ratio; α-Toc/Chl, α-tocopherol per unit of Chl. Genotypes: Rooted cuttings, “Chemlali” and “Chetoui”; Seedlings, “Chemlali S,” “Chetoui S,” “Zarrazi S” and “Oleaster S.”

A summary of the differences in the response to severe water stress among olive trees are shown in Figure 6. Our data suggest that “Zarrazi S” may cope better with drought conditions which exhibited a lower decrease in growth and net photosynthesis rates, as well as a higher accumulation of photoprotective molecules. “Chemlali” rooted cuttings showed an acclimation response close to that of “Zarrazi S.” “Oleaster S” can be considered the most sensitive to these water stress conditions as it was the genotype showing the highest depressive effect of water stress on growth and net photosynthesis rates and lower photoprotective capacity. In susceptibility to water stress, “Oleaster S” were followed by “Chemlali S,” “Chetoui S” and “Chetoui” rooted cuttings (Figure 6). To our knowledge, this is the first time in which the activation of photo- and antioxidant protection mechanisms is linked to leaf water status, net photosynthetic rates and chlorophyll loss in olive trees, showing that mechanisms of photo- and antioxidant protection can still be active at very low water potentials in this plant species and that the better adapted genotypes show their photo- and antioxidant protection capacity intact, despite suffering from severe drought. In general, “Chemlali” rooted cuttings were more drought tolerant than “Chemlali S”, and “Chetoui” root cuttings and seedlings did respond similarly, thus indicating that vegetative propagation in these two genotypes, and most particularly in Chemlali, may be effective in large-scale propagation for field cultivation in arid or semi-arid areas. Further research is, however, needed to unravel whether the same occurs in “Zarrazi” genotype, which was the genotype showing the highest drought stress tolerance among all the genotypes tested.

In conclusion, withholding irrigation for 60 days induced a considerable decrease in growth, leaf water potential and net photosynthesis rates in 1-year-old olive trees. This might lead to the activation of photoprotective mechanisms, including, increases in xanthophyll cycle de-epoxidation, and accumulation of α-tocopherol as chlorophyll loss progressed in severely stressed plants. These increases may contribute to the survival of young olive trees under severe water stress conditions. The study also revealed that “Zarrazi” seedlings may serve as drought-tolerant rootstock which exhibited a lower decrease in growth and net photosynthesis rate, as well as a higher antioxidant activity. “Oleaster,” “Chemlali” and “Chetoui” seedlings could be comparatively considered in this order as more drought-sensitive rootstocks. “Chemlali” rooted cuttings showed the tolerance behavior closer to that of “Zarrazi” seedlings, thus, “Chemlali” rooted cuttings are interesting to create vegetatively propagated drought-tolerant rootstocks and/or cultivars used for breeding programs.

## Data Availability Statement

The raw data supporting the conclusions of this article will be made available by the authors, without undue reservation.

## Author Contributions

SB, AC-R, and SM-B conceived and designed the study. SB, EF, and MM performed the experiments. SB, OE, and SM-B analyzed the data and wrote the manuscript, while other authors performed a critical revision of the manuscript. AC-R, FBA, LF, and SM-B supervised the work. All authors approved the final manuscript.

## Conflict of Interest

The authors declare that the research was conducted in the absence of any commercial or financial relationships that could be construed as a potential conflict of interest.

## Abbreviations

Ψ _*L*_: leaf water potential
^1^O_2_: singlet oxygen
A_*n*_: net photosynthesis rate
Chl: chlorophyll
DPS: de-epoxidation state of the xanthophyll cycle
DW: dry weight
LPR: leaf production rate
PSII: photosystem II
ROS: reactive oxygen species
SGR: stem growth rate
VZA: xanthophyll cycle pool.
